# Developing a Spatial-Skills-Focused Music Program for Older Adults With Changes in Cognition

**DOI:** 10.1093/geroni/igaa057.934

**Published:** 2020-12-16

**Authors:** Jennie Dorris, Juleen Rodakowski

**Affiliations:** University of Pittsburgh, Pittsburgh, Pennsylvania, United States

## Abstract

Older adults with cognitive changes need stimulating programming to maximize their cognitive abilities. One area to maximize includes spatial skills, its decline can lead to disorientation and wandering. Music has potential to maximize spatial skills: reading music’s notation is associated with enhanced spatial skills in children and professional musicians. It’s critical to understand the potential impact of a spatially focused music program for older adults with changing cognition; if successful, future music programs could support people staying orientated in their environments and living independently longer. We developed and assessed a six-week marimba program focused on reading music with 15 older adults ages 65-89 with changes in cognition. We compared their scores on the Orientation Test from the Test of Visual Perceptual Skills pre- and post-intervention and assessed if participants self-selected to read music notation. Participants scored an average Modified Mini Mental State Examination (3MSE) score of 81.3 (SD = 11.0). On average, participants’ scores on the Orientation Test moved from 13.4 (SD =1.9) to 14.1 (SD= 2.7), providing a cohen’s d effect size of 0.3. Over the six weeks, 11 out of the 15 participants selected to read music for at least one class, indicating a statistically significant change using the Wilcoxon signed-rank test (Z = -3.16, p < 0.01), suggesting that older adults with cognitive changes may be able to learn to read music. This is important, as a spatially focused music program may maximize spatial skills that older adults need to successfully navigate their world safely and independently.

